# Prehabilitation—A Simple Approach for Complex Patients: The Results of a Single-Center Study on Prehabilitation in Patients with Ovarian Cancer Before Cytoreductive Surgery

**DOI:** 10.3390/cancers16234032

**Published:** 2024-12-01

**Authors:** Marcin Adam Zębalski, Aleksandra Krzywon, Krzysztof Nowosielski

**Affiliations:** 1 Department of Gynecology, Obstetrics and Gynecological Oncology, University Clinical Center of the Medical University of Silesia, 40-752 Katowice, Poland; ginpol@uck.katowice.pl; 2 Department of Biostatistics and Bioinformatics, Maria Sklodowska-Curie National Research Institute of Oncology, Gliwice Branch, 44-102 Gliwice, Poland

**Keywords:** prehabilitation, ovarian cancer, gynecological oncology

## Abstract

**Background/Objectives**: Prehabilitation is a low-cost, safe procedure with no side effects, and it may have a positive impact on postoperative outcomes. However, it is not widely implemented. Our study aimed to assess the impact of prehabilitation on postoperative outcomes in patients with ovarian cancer within the field of gynecological oncology. **Methods**: We analyzed 110 patients with ovarian cancer who participated in a prehabilitation program before cytoreductive surgery. Based on the results of a 6-min walk test (6MWT), patients were divided into two groups: Group A (patients who improved their 6MWT results) and Group B (patients who did not improve their 6MWT results). **Results**: Patients in Group A demonstrated better postoperative outcomes. The length of hospital stay was significantly shorter in Group A compared to Group B (median 7 [5, 9] vs. 9 [6, 17], *p* = 0.032). Group A also had a lower overall number of complications and also fewer complications, as summarized by the Clavien–Dindo classification, compared to Group B. **Conclusions**: Patient adherence to prehabilitation recommendations was adequate. Prehabilitation was associated with improved postoperative outcomes, including shorter hospital stays and fewer complications. These benefits were more pronounced with higher patient compliance with the prehabilitation program and improvements were recorded in preoperative physical capacity.

## Simple Summary

Properly conducted multimodal prehabilitation can reduce the side effects of major surgical procedures. In recent years, prehabilitation has been increasingly implemented, though there remains a research gap in its application within gynecological oncology. The aim of our study was to assess the impact of prehabilitation on postoperative outcomes in patients with ovarian cancer. Furthermore, we investigated whether postoperative outcomes differed between patients who improved their physical fitness through prehabilitation and those who did not. Nutritional interventions were also undertaken, with subsequent assessments of changes in laboratory parameters. We found that improved preoperative physical fitness is associated with better postoperative outcomes, including fewer complications and shorter hospital stays.

## 1. Introduction

Ovarian cancer is a systemic disease that is primarily treated with surgery, followed by chemotherapy. In cases of advanced disease, treatment may begin with neoadjuvant chemotherapy, after which patients are referred for delayed cytoreductive surgery (IDS—interval debulking surgery). In both treatment pathways, it is recommended that patients undergo a prehabilitation program to prepare for treatment [1].

In recent years, there has been a significant increase in data on the impact of prehabilitation on postoperative outcomes. An increasing number of clinicians worldwide are adopting this affordable, safe, and effective preoperative care protocol for patients awaiting surgery.

Prehabilitation is a multimodal process that combines physical and mental preparation for surgery. Physical preparation includes aerobic and resistance activities of increasing intensity, along with a high-protein diet supplemented with anti-inflammatory and immunomodulatory agents, such as omega-3 fatty acids, beta-glucan, eicosapentaenoic acid (EPA), and docosahexaenoic acid (DHA). The preoperative optimization of laboratory results involves managing anemia, blood glucose, and thyroid levels, electrolyte balance, and vitamin D deficiency. Mental preparation is equally essential; high-risk patients and those showing symptoms of depression should be identified early in prehabilitation and provided with appropriate treatment and psychological or psychiatric support [1,2,3,4]. Recently, the concept of cognitive prehabilitation has emerged, aiming to reduce postoperative cognitive disorders [5].

Numerous studies have shown that incorporating prehabilitation into preoperative care positively impacts postoperative outcomes. It has been associated with fewer complications and shorter hospital stays [6,7,8,9,10]. Moreover, prehabilitation in ovarian cancer patients has been linked to a shorter time until chemotherapy initiation, potentially impacting overall survival outcomes [11]. For patients awaiting interval debulking surgery after neoadjuvant chemotherapy, improvements in various health parameters may contribute to enhanced postoperative recovery [12].

Despite numerous reports on the positive impact of prehabilitation on perioperative and postoperative outcomes, there is still no clear consensus on its use in preoperative care, particularly within gynecologic oncology. Furthermore, large, multicenter, randomized studies that could establish prehabilitation as a standard component of preoperative care are lacking [13].

This study aims to assess the effectiveness of preoperative prehabilitation on postoperative outcomes in patients with ovarian cancer. We also seek to explore the relationship between the quality of prehabilitation preparation and patient engagement in the process. To this end, we compared the prehabilitated patients, dividing them into two groups based on their 6MWT results, and evaluated how preoperative physical capacity relates to postoperative outcomes.

## 2. Materials and Methods

### 2.1. Patients

The study comprised patients at the Department of Gynecology, Obstetrics and Gynecological Oncology in Katowice, Poland, who were scheduled for surgery due to ovarian cancer or suspected ovarian tumors between April 2021 and May 2024. All patients referred for surgery were enrolled in the prehabilitation program, with the following exclusion criteria: lack of consent to participate in the study, an ECOG performance status score of >2, or severe neurological disorders preventing comprehension of and adherence to recommendations. Patients whose ovarian cancer was not confirmed by histopathological examination were excluded from further analysis.

### 2.2. Statistical Analysis

Categorical variables were summarized as frequencies and percentages, while continuous data were presented as median values with interquartile ranges (IQR, 25% to 75%). Differences between the two groups were assessed using the Wilcoxon rank-sum test for continuous variables and Fisher’s exact test for categorical variables. Changes in the measured parameters between two time points were evaluated using the one-sample Wilcoxon signed-rank test. Odds ratios (ORs) with 95% confidence intervals (CIs) were estimated using univariate and multivariate logistic regression models. Variables with a *p*-value of less than 0.05 in the univariate analysis were included in the multivariate analysis. A two-sided *p*-value of less than 0.05 was considered statistically significant. All computational analyses were conducted in the R environment for statistical computing, using version 4.0.1, “See Things Now”, released on 6 June 2020 (R Foundation for Statistical Computing, Vienna, Austria, http://www.r-project.org, accessed on 20 December 2022).

### 2.3. Study Protocol

After excluding disqualifying factors from the study, all the remaining patients referred to the Department of Gynecology, Obstetrics, and Gynecological Oncology were recommended to participate in a prehabilitation program as preparation for surgery. During the initial visit, the patient was assessed by the physician overseeing the prehabilitation program and a physiotherapist. This assessment included a comprehensive subjective and objective examination, as well as evaluations of physical fitness, nutritional status, the occurrence and/or severity of anxiety and depression, and a frailty scale. The prehabilitation program was conducted independently at home, with remote supervision by the prehabilitation team. If a consultation were needed, the patient could come to the clinic for further assistance. The day before surgery, the patient was admitted to the clinic for laboratory tests and a repeat overall assessment.

#### 2.3.1. Patient Assessment

During the first visit, each patient underwent an initial assessment. This assessment included a basic medical examination, a review of the patient’s medical history, and an evaluation of vital signs. Physical fitness was assessed using the 6-min walk test (6MWT) and the calculated maximum oxygen consumption (VO^2^max). Nutritional status was determined through an interview with the patient and the results of nutritional status questionnaires, specifically the mini nutritional assessment (MNA). The occurrence and severity of anxiety and depression were evaluated using the Hospital Anxiety and Depression Scale (HADS) questionnaire. Frailty was assessed using the G8 geriatric screening scale.

#### 2.3.2. Prehabilitation Intervention

Based on the initial assessment, each patient was provided with a preoperatively home-based exercise program that included a 10-min warm-up, aerobic training, resistance training, breathing exercises, and stretching. The exercise program was developed by the Prehabilitation Team and was standardized for all patients; however, the intensity, duration of resistance training, and number of repetitions were individually tailored to each patient, based on the initial assessment, the 6MWT, and VO^2^max measurements.

Each patient was advised to follow a high-protein diet, with a recommended protein intake of 1.5 to 2.0 g per kilogram of body weight. Simultaneously, patients were encouraged to limit their carbohydrate intake, particularly simple carbohydrates. A high-protein diet was recommended according to current preoperative care guidelines to replenish protein deficiencies, increase muscle anabolism, increase muscle strength, and increase the synthesis of new proteins, including the production of antibodies and proteins responsible for the healing process [1]. We recommended the limiting of carbohydrates, especially simple carbohydrates, in order to maximize the dietary focus on protein and normalize carbohydrate metabolism. Patients received recommendations for using protein supplements, including those with immunomodulatory and anti-inflammatory properties (e.g., omega-3 fatty acids, beta-glucan, eicosapentaenoic acid (EPA), and docosahexaenoic acid (DHA)). Patients suspected of malnutrition, indicated by a score of two or more points on the Malnutrition Universal Screening Tool (MUST), were referred for specialist advice from a clinical dietitian.

Those who scored 8 or more points on the Hospital Anxiety and Depression Scale (HADS) questionnaire were referred to a psychologist. If psychological care proved insufficient, those patients were subsequently referred to a psychiatrist.

Patients who smoked cigarettes or consumed alcohol excessively were encouraged to stop using these substances, with active counseling provided on the harmful effects of nicotine and alcohol on surgical outcomes.

Before the initial visit, each patient underwent laboratory tests, including a complete blood count, electrolyte panel, liver function tests, creatinine levels, thyroid parameters, total protein, albumin, and vitamin D concentrations. If any abnormalities were detected, we initiated pharmacological treatment for specific deficiencies and optimized the laboratory test results.

### 2.4. Surgery

Each patient included in the study was diagnosed with ovarian cancer and was then referred for surgical treatment. The patients underwent cytoreductive surgery via a laparotomy. The study group comprised patients referred for primary debulking surgery, interval debulking surgery, or secondary debulking surgery for ovarian cancer.

All patients received standard perioperative care according to enhanced recovery after surgery (ERAS) recommendations [14]. The day before surgery, patients were given protein supplements, along with a high-carbohydrate meal (carbo-loading) and psychological care. On the day of surgery, premedication was minimized, and a high-carbohydrate drink was administered two hours prior to the procedure. Carbohydrate loading was recommended to limit catabolism during surgery. In the early postoperative period, appropriate pain relief was provided, and patients were encouraged to mobilize on the same day as the procedure. In the following days, postoperative rehabilitation was implemented, promoting physical activity and aiming for the prompt removal of drains (on the first day) and the Foley catheter (on the second day).

## 3. Results

### 3.1. Patients

We recruited 110 patients with confirmed ovarian cancer for the study. Among these, 80 patients (72.7%) underwent primary cytoreductive surgery, 18 patients (16.4%) underwent interval debulking surgery, and 12 patients (10.9%) underwent secondary cytoreduction. All patients included in this analysis participated in a prehabilitation program with a median duration of 17 days (IQR: 13–23 days) and a mean ± SD of 21.4 ± 18.6 days. Detailed characteristics of the patients are presented in Table 1.

### 3.2. Physical Fitness

The median 6-min walk test (6MWT) distance increased by 7.0 m (IQR: −7.0, 27.0) at *p* < 0.001, while the VO^2^max increased by a median of 0.16 mL/kg^−1^⋅min^−1^ (IQR: −0.16, 0.62) at *p* < 0.001 for all patients. Among the entire group, 62 patients (56.4%) improved their 6MWT result by a median of 23.5 m (IQR: 10, 40). Of the remaining patients, 16 patients (14.5%) recorded the same 6MWT result, while 32 patients (29.1%) recorded a worse 6MWT result on the day of hospital admission compared to their performance at the beginning of prehabilitation.

We divided the patients into two groups, based on the 6MWT results: group A (62 patients [56.4%]) included those who improved their 6MWT distance during the prehabilitation period, while group B (48 patients [43.6%]) comprised those whose 6MWT distance did not change or deteriorated. The median duration of prehabilitation was 17.5 (IQR: 13–26), mean ± SD: 24.3 ± 22.8 days in group A, compared to the median of 15.5 (IQR: 12–20.3), mean ± SD: 17.7 ± 10.2 days in group B. The values of 6MWT and VO^2^max, along with their changes in both groups, are shown in Table 2.

Patients whose physical fitness, as measured by the 6-min walk test (6MWT), improved during prehabilitation constituted a larger portion of the group (56.4%) and were statistically similar to the patients whose physical fitness did not improve (43.6%). Other variables, such as age, body mass index (BMI), menopause status, education level, fitness level, professional activity, smoking status, and the presence of comorbidities, were comparable between both groups. Moreover, the percentage of patients declaring physical activity in the preoperative period was similar (24% vs. 23%, *p* > 0.05). Detailed characteristics of the patients divided into groups are presented in Table 3.

### 3.3. Surgery

All patients underwent cytoreductive surgery for ovarian cancer, with all procedures performed via a laparotomy. Groups A and B were comparable in terms of the duration of surgery, type of cytoreduction, radicality of surgery, and cancer stage. Both groups were also comparable regarding the surgical procedures performed, except for the frequency of splenectomy and pelvic peritonectomy procedures. Splenectomies and pelvic peritonectomies were performed significantly more often in group A (14% vs. 2%, *p* = 0.007 and 33% vs. 14%, *p* = 0.013, respectively). Other surgical procedures, as well as the frequency of intestinal anastomoses and stoma formation, were similar in both groups (*p* > 0.05). A detailed comparison of the surgical details and procedures performed in both groups is presented in Table 4 and Table 5.

### 3.4. Laboratory Results

We found no differences in blood count or nutritional parameters during the prehabilitation program. Despite the recommendation of a high-protein diet, along with supplementation of protein preparations containing anti-inflammatory and immunomodulatory additives, we did not observe an increase in total protein and albumin concentrations during prehabilitation. The only laboratory parameter that significantly improved during this period was vitamin D concentration, with a median difference of 10 ng/mL (IQR: 4, 17), *p* < 0.001. Detailed changes in the laboratory parameter values before and at the end of prehabilitation are presented in Table 6.

When comparing the results of the laboratory tests—including hemoglobin concentration, red blood cell count (RBC), white blood cell count (WBC), and C-reactive protein level (CRP)—which were performed at the end of hospitalization after surgery, we found no significant differences between the two groups (*p* > 0.05). We also compared the differences in laboratory test results from the day of admission to the hospital and the day of discharge to home between group A and group B, but did not observe a significant difference in WBC, RBC, hemoglobin (HGB), or CRP values (*p* > 0.05).

### 3.5. Complications

**Figure 1:**
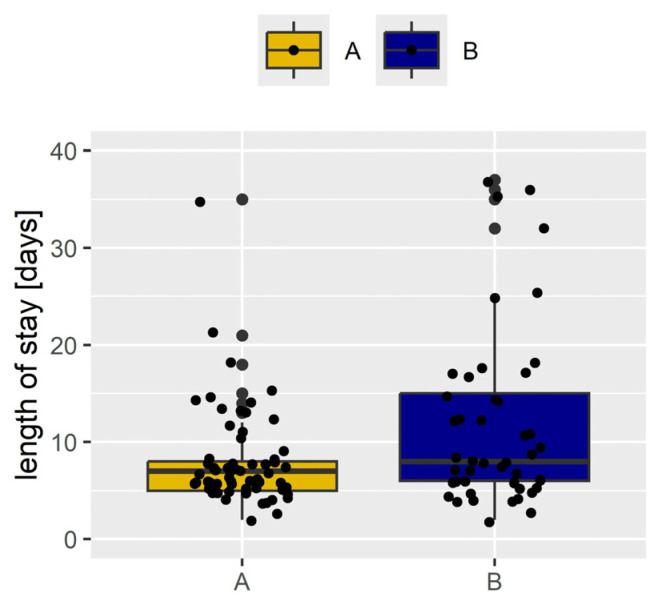
Comparison of the length of stay after surgery in both groups.

With regard to group A, we demonstrated a significant reduction in the length of stay after the procedure—median 7 days (IQR: 5, 9) vs. 9 days (IQR: 6, 17) in group B; *p* = 0.032 (Figure 1). The frequency of serious complications, such as the need for reoperation, eventration, and hospitalization in the intensive care unit due to circulatory, respiratory, or cardiopulmonary failure, or patient death, did not differ significantly between the two groups. However, the frequency of serious complications classified as grade 3 or higher according to the Clavien–Dindo classification was significantly higher in group B (16 [33.3%] vs. 8 [12.9%], *p* = 0.02).

With regard to group B, significantly more frequent complications included improper wound healing and wound dehiscence (1.6% vs. 13%, *p* = 0.042), the need for vacuum therapy (1.6% vs. 13%, *p* = 0.042), the need for blood transfusion (23% vs. 42%, *p* = 0.032), the need to transfer the patient to another center for further treatment (0% vs. 10%, *p* = 0.014), and the modification of antibiotic therapy due to a lack of response to the initial standard antibiotic treatment (8.1% vs. 25%, *p* = 0.015). Other complications did not differ statistically significantly between the two groups. We did not observe a difference in readmission rates between groups A and B (2 [3.2%] vs. 2 [4.2%], *p* > 0.9). All complications are presented in Table 7.

Despite the lower number of complications in group A, we did not observe any significant differences between groups A and B regarding the time from surgery to the initiation of adjuvant treatment. However, there was a trend toward a shorter time until the initiation of adjuvant chemotherapy in group A, with a median of 56 days (IQR: 49, 81) compared to 68 days (IQR: 57, 80) in group B (*p* = 0.4).

We performed a multiple logistic regression analysis, demonstrating that both the duration of surgery and physical fitness directly impacted postoperative complications. Our findings indicated that with each additional 30 min of surgery, the risk of complications increased by 1.44 times. Furthermore, in patients whose physical fitness did not improve during prehabilitation, the risk of postoperative complications increased by 3.31 times (Table 8).

## 4. Discussion

The primary objectives of this study were to assess the effects of prehabilitation on the incidence of postoperative complications, the length of hospital stay following cytoreductive surgery, and the time until the initiation of adjuvant chemotherapy. We recommended a multimodal prehabilitation program to all patients included in this study prior to their cytoreductive surgery for ovarian cancer. The secondary objective was to evaluate the quality of prehabilitation preparation, for which we utilized the 6-min walk test (6MWT) and VO^2^max measurements. Based on these assessments, we divided patients into two groups according to their improvement or lack of improvement in preoperative physical fitness as a result of the prehabilitation intervention, allowing us to compare postoperative outcomes between the groups.

Our study confirms the positive impact of prehabilitation on preparing patients for cytoreductive surgery due to advanced ovarian cancer and the associated postoperative outcomes. We demonstrated a significant increase in physical capacity, as measured by the 6MWT and VO^2^max, in the patients included in this study. By separating patients who showed improvement in their 6MWT results, we divided the patients into two groups based on these results. This analysis revealed that patients who significantly improved their physical capacity (group A) had a shorter hospital stay after surgery and experienced fewer moderate complications. Their improved performance in the 6MWT and their VO^2^max levels indicate that patients adhered to the prehabilitation recommendations, confirming their compliance. We did not observe differences in serious complications such as death, intensive care unit hospitalization, or the need for reoperation.

In contrast, group B, consisting of 48 patients, did not achieve an improvement in physical fitness; in fact, we noted a decrease in fitness levels. Several factors may contribute to this outcome. One possibility is that these patients failed to adhere to the prehabilitation recommendations. Additionally, significant limiting factors for patients with ovarian cancer may include advanced age, the severity of neoplastic disease, pain, or the presence of increasing ascites, all of which can hinder adherence to a training plan or a proper diet. An important factor influencing adherence to prehabilitation recommendations may be the patient’s mental state. Although those patients with symptoms of depression and severe anxiety were referred to psychological care preoperatively, this is undoubtedly an important factor limiting preoperative preparation.

Our results align with the majority of the existing literature regarding the impact of prehabilitation on postoperative outcomes. In recent years, there has been a substantial increase in studies examining prehabilitation. These studies indicate a reduction in both the length of hospital stay after surgery [11,15,16,17] and the incidence of complications [8,18,19,20], with some studies reporting improvements in both areas [9,11,21,22,23,24,25]. A meta-analysis by Skorepa et al., which included 16 studies—6 of which were randomized—found that prehabilitation not only shortens hospital stays but is also associated with a reduction in serious complications by up to 44% [10].

In the field of gynecological oncology, prehabilitation shows promising results. For instance, a study by Li et al. assessed the effectiveness of a short-term prehabilitation program (mean intervention time of 7.2 days) for patients with gynecological cancers, demonstrating an improvement in overall fitness as measured by the 6MWT. This improvement translated into better fitness results at 30 days post-surgery in the prehabilitation group. Additionally, Li et al. noted a reduction in anxiety levels, as measured by the Hospital Anxiety Scale (HAS) [26]. Similarly, a study by Lee et al. evaluated the impact of prehabilitation on patients with endometrial cancer, finding that the intervention improved cardiorespiratory fitness, overall health, and increased muscle strength. However, neither of these studies assessed the influence of prehabilitation on the frequency and quality of postoperative complications [26,27]. Moreover, Diaz-Feijoo et al. reported that patients undergoing prehabilitation experienced a shorter hospitalization time (5 days in the prehabilitation group versus 7 days in the control group), but the authors did not find differences in the frequency of complications [11].

The available data also suggest improvements in laboratory parameters during prehabilitation preparation. Hypoalbuminemia has been directly associated with a higher risk of postoperative complications and prolonged hospital stays [28,29,30]. Similarly, reduced preoperative hemoglobin levels correlate with an increased risk of complications [31,32]. Diaz-Feijoo demonstrated an increase in preoperative albumin concentrations among patients undergoing prehabilitation due to nutritional interventions (0.235 g/L vs. 0.180 g/L, *p* = 0.007) [11]. A study by Kabata et al. found that the control group experienced a significant decrease in albumin levels during the preoperative period, whereas patients receiving appropriate supplementation showed an increase in albumin values [33]. Furthermore, Foley et al. highlighted that severe anemia is common among patients with gynecological cancers, particularly ovarian cancer, and is associated with a significantly increased risk of complications. The proper detection and treatment of anemia are crucial for improving surgical outcomes [34].

We observed a lower number of complications, both overall and when classified according to the Clavien–Dindo system, in the group of patients who experienced improvements in physical fitness (group A). Notably, the overall frequency of complications among all patients included in the prehabilitation program was generally low. According to the available data, the incidence of serious complications classified as Clavien–Dindo 3A and above in major abdominal surgeries can be as high as 30% [35,36,37]. In our study, the overall percentage of serious complications in the entire study group was 21.8%.

While we did not find a significant difference in the time until the initiation of adjuvant chemotherapy between the two groups, other studies have indicated that prehabilitation can shorten the time from surgery to the commencement of adjuvant chemotherapy [11,13]. This finding is particularly important, as it is widely recognized that delays in starting adjuvant chemotherapy for ovarian cancer patients can significantly worsen prognosis [38]. Conversely, similar to our results, Trepanier et al. reported no significant difference in the time to adjuvant chemotherapy between the prehabilitation group and the group receiving standard preoperative care [14].

However, there are studies that indicate no positive effect of prehabilitation. Based on the meta-analysis prepared by Waterland et al., it was found that prehabilitation is associated with a shortened hospital stay but does not affect the incidence of postoperative complications [7]. According to the study by van der Hulst et al., prehabilitation had no effect on the incidence of postoperative complications [39]. Another study that showed no impact of prehabilitation on postoperative complications and the length of hospital stay is a retrospective analysis prepared by Pang et al. [40]. Similarly, a study by Karlsson et al. demonstrated no significant reduction in the frequency of postoperative complications or length of hospital stay in the prehabilitation group compared to the control group [41]. In a randomized study by Dronkers et al. that assessed the effect of inspiratory muscle training on the incidence of complications following abdominal surgery, no significant difference was found between the prehabilitation and control groups [42].

It should be emphasized that prehabilitation is a safe approach in preoperative preparation. However, this does not mean that it is an easy procedure. Proper prehabilitation preparation requires a great deal of commitment from both the patient and the person responsible for the patient’s care during the preparatory process for it to be successful. Unimodal prehabilitation programs have been described, but greater benefits are obtained from multimodal programs [43]. The multimodal approach requires even greater involvement from the prehabilitation team. Only a combination of all elements of prehabilitation allows for achieving success, which translates into better postoperative results, including shorter hospitalization times, fewer complications, better patient well-being, and a shorter period until starting adjuvant treatment. In turn, better postoperative outcomes, such as a shorter period until the initiation of adjuvant treatment, may translate into improved long-term outcomes, resulting in better overall survival and progression-free survival.

This study is limited by its retrospective nature and the limited time available for the prehabilitation intervention. Home-based exercises also seem to be less effective than supervised interventions. Our intervention was based on home exercises, while there are other authors who conducted supervised prehabilitation programs [44,45]. It can be said that the implementation of prehabilitation programs at home is not as efficient as supervised prehabilitation programs. The patients included in our study are oncology patients with many limitations, such as older age, the presence of comorbidities, and various health burdens such as increasing ascites, which are characteristic of ovarian cancer and which may hinder the proper implementation of the prehabilitation program. Selecting the most needful and most burdened patients and providing them with individual care throughout the entire period of prehabilitation seems to increase the degree of compliance with appropriate prehabilitation recommendations and, thus, improve the quality of preoperative preparation.

## 5. Conclusions

In our study, patients adhered to prehabilitation recommendations, resulting in improved physical capacity, as evidenced by enhancements in the 6-min walk test (6MWT) and VO^2^max measurements. Prehabilitation is associated with a reduction in postoperative complications and a shorter hospital stay following cytoreductive surgery for ovarian cancer. Notably, patients who achieved better preoperative physical capacity after adhering to multimodal prehabilitation protocols experienced fewer postoperative complications and shorter hospital stays compared to those whose physical capacity did not improve. However, we did not observe a significant reduction in the delay until the initiation of adjuvant treatment. To optimize outcomes, well-designed randomized trials with larger sample sizes are warranted. Given these findings, further large-scale studies on prehabilitation in the context of gynecological oncology are essential.

## Figures and Tables

**Table 1:** General characteristics of all patients included in the study.

Characteristic	Value (*n* Total = 110)
Age (years), median [IQR]	65 (51, 69)
BMI (kg/m^2^), median [IQR]	26.4 (24.1, 29.7)
City inhabitant, *n*/*n* total (%)	93 (85)
Menopause, *n*/*n* total (%)	83/110 (75)
Nulliparous, *n*/*n* total (%)	11/110 (10)
Tertiary education, *n*/*n* total (%)	36/110 (33)
Working patients, *n*/*n* total (%)	41/110 (37)
Smokers, *n*/*n* total (%)	23/110 (21)
Hypertension, *n*/*n* total (%)	61/110 (55)
Ischemic heart disease, *n*/*n* total (%)	17/110 (15)
Diabetes, *n*/*n* total (%)	14/110 (13)
Hypothyroidism, *n*/*n* total (%)	17/110 (15)
Asthma, *n*/*n* total (%)	3/110 (2.7)
Chronic obstructive pulmonary disease, *n*/*n* total (%)	2/110 (1.8)
ECOG 0, *n*/*n* total (%)	88/110 (80)
Physical activity *, *n*/*n* total (%)	30/110 (27.3)
CA-125 (U/mL), median [IQR] **	187 (58, 1140)
Hemoglobin (g/dL), median [IQR] **	12.6 (11.6, 13.7)
RBC (×10^6^/μL), median [IQR] **	4.4 (4.0, 4.7)
WBC (×10^3^/μL), median [IQR] **	7.6 (6.0, 9.5)
Albumin concentration (g/dL), median [IQR] **	4.4 (4.2, 4.7)
Total protein concentration (g/dL), median [IQR] **	7.3 (7.0, 7.6)
Creatinine (mg/dL), median [IQR] **	0.8 (0.7, 0.9)
Vitamin D concentration (ng/mL), median [IQR] **	25 (13, 36)

Abbreviations: IQR—interquartile range, BMI—body mass index, ECOG—Eastern Cooperative Oncology Group. *—A patient was considered physically active if she reported regularly performing at least 150 min of moderate-intensity activity per week. **—Parameters were measured at the beginning of the prehabilitation program.

**Table 2:** Changes in 6MWT and VO^2^max values during the prehabilitation period.

Characteristic	Group A (*n* = 62)				Group B (*n* = 48)			
	Introductory Visit	Day of Admission to Hospital	Change *	*p*-Value	Introductory Visit	Day of Admission to Hospital	Change *	*p*-Value
6MWT [m], median [IQR]	480.0 (440.0, 520.0)	506.0 (450.0, 547.0)	24 (10, 40)	<0.001	474.0 (420.0, 515.0)	460.0 (420.0, 502.5)	−10 (−17.5, 0)	<0.001
VO^2^max [mL/kg^−1^⋅min^−1^], median [IQR]	27.6 (23.5, 33.4)	28.0 (24.6, 33.7)	0.56 (0.23, 0.92)	<0.001	25.6 (22.0, 30.7)	25.2 (21.7, 30.5)	−0.23 (0.40, 0.00)	<0.001

Abbreviations: 6MWT—6-min walk test. VO^2^max—maximum oxygen consumption. *—Change was defined as the difference in 6MWT or VO^2^max measures between the day of hospital admission for surgery and the initial visit.

**Table 3:** General characteristics of both groups of patients.

Characteristic	Group A (*n* Total = 62)	Group B (*n* Total = 48)	*p*-Value
Age (years), median [IQR]	65 (53, 68)	64 (51, 69)	0.7
BMI (kg/m^2^), median [IQR]	26.0 (23.0, 29.0)	27.0 (24.2, 30.8)	0.08
City inhabitant, *n*/*n* total (%)	48/62 (77.4)	45/48 (93.7)	0.03
Menopause, *n*/*n* total (%)	48/62 (77.4)	35/48 (72.9)	0.66
Nulliparous, *n*/*n* total (%)	2/62 (3.2)	9/48 (18.7)	0.01
Tertiary education, *n*/*n* total (%)	20/62 (32.3)	16/48 (33.3)	1.00
Working patients, *n*/*n* total (%)	21/62 (33.9)	20/48 (41.7)	0.43
Smokers, *n*/*n* total (%)	11/62 (17.7)	12/48 (25)	0.48
Hypertension, *n*/*n* total (%)	32/62 (51.6)	29/48 (60.4)	0.44
Ischemic heart disease, *n*/*n* total (%)	8/62 (13.0)	9/48 (18.7)	0.44
Diabetes, *n*/*n* total (%)	9/62 (14.5)	5/48 (10.4)	0.58
Hypothyroidism, *n*/*n* total (%)	12/62 (19.3)	5/48 (10.4)	0.29
Asthma, *n*/*n* total (%)	3/62 (4.8)	0/48 (0)	0.26
Chronic obstructive pulmonary disease, *n*/*n* total (%)	0/62 (0)	2/48 (4.2)	0.19
ECOG 0, *n*/*n* total (%)	53/62 (85.5)	35/48 (73.0)	0.15
Physical activity *, *n*/*n* total (%)	15/62 (24.2)	11/48 (22.9)	1.00
CA-125 (U/mL), median [IQR] **	304 (64, 1312)	150 (49, 855)	0.3
Hemoglobin (g/dL), median [IQR] **	12.6 (11.5, 13.7)	12.8 (11.6, 13.6)	0.9
RBC (×10^6^/µL), median [IQR] **	4.4 (3.8, 4.0)	4.4 (4.0, 4.8)	0.4
WBC (×10^3/^µL), median [IQR] **	7.0 (4.8, 9.0)	8.2 (7.0, 10.4)	0.006
Albumin concentration (g/dL), median [IQR] **	4.5 (4.2, 4.7)	4.4 (4.2, 4.7)	0.5
Total protein concentration (g/dL), median [IQR] **	7.3 (7.0, 7.6)	7.3 (7.0, 7.6)	0.9
Creatinine (mg/dL), median [IQR] **	0.80 (0.70, 0.90)	0.75 (0.70, 0.80)	>0.9
Vitamin D concentration (ng/mL), median [IQR] **	26.4 (17.4, 37.6)	21.4 (15.1, 33.2)	0.08

Abbreviations: IQR—interquartile range, BMI—body mass index, ECOG—eastern cooperative oncology group, and VO^2^max—maximum oxygen consumption. *—A patient was considered physically active if she reported regularly performing at least 150 min of moderate-intensity activity per week. **—Parameters were measured at the beginning of the prehabilitation program.

**Table 4:** Surgical characteristics of patients in group A and group B.

Characteristic	Group A (*n* Total = 62)	Group B (*n* Total = 48)	*p*-Value
Duration of surgery (min), median [IQR]	258 (210, 334)	250 (164, 340)	0.4
Type of surgery			
Primary debulking surgery, *n*/*n* total (%)	44/62 (71)	36/48 (75)	0.6
Interval debulking surgery, *n*/*n* total (%)	12/62 (19)	6/48 (13)	
Secondary cytoreduction, *n*/*n* total (%)	6/62 (9.7)	6/48 (13)	
Residual disease			
R0, *n*/*n* total (%)	55/62 (88.7)	39/48 (81.3)	0.4
R1, *n*/*n* total (%)	5/62 (8.1)	4/48 (8.3)	
R2, *n*/*n* total (%)	2/62 (3.2)	5/48 (10.4)	
Aletti complexity score, median (IQR)	4.0 (2.0, 8.0)	4.0 (2.0, 6.5)	0.3
Low complexity score (score ≤ 3), *n*/*n* total (%)	24/62 (38.7)	19/48 (39.6)	>0.9
Intermediate complexity score (score 4–7), *n*/*n* total (%)	21/62 (33.9)	17/48 (35.4)	>0.9
High complexity score (score ≥ 8), *n*/*n* total (%)	17/62 (27.4)	12/48 (25)	0.8
PCI, median (IQR)	10.0 (4.0, 19.0)	11.5 (4.75, 16.5)	>0.9
Low-grade carcinoma, *n*/*n* total (%)	14/62 (23)	10/48 (21)	1.0
High-grade carcinoma, *n*/*n* total (%)	48/62 (77)	38/48 (79)	1.0
FIGO stages			
FIGO 1, *n*/*n* total (%)	11/62 (18)	7/48 (15)	0.4
FIGO 2, *n*/*n* total (%)	1/62 (1)	3/48 (6)	
FIGO 3, *n*/*n* total (%)	44/62 (71)	36/48 (75)	
FIGO 4, *n*/*n* total (%)	6/62 (10)	2/48 (4)	

Abbreviations: IQR—interquartile range, PCI—peritoneal cancer index.

**Table 5:** Detailed comparison of surgical procedures performed in both groups during surgery.

Characteristic	Group A (*n* Total = 62)	Group B (*n* Total = 48)	*p*-Value
Radical hysterectomy with bilateral salpingo-oophorectomy, *n*/*n* total (%)	55/62 (89)	39/48 (81)	0.3
Fertility preserving surgery, *n*/*n* total (%)	0/62 (0)	2/48 (4.2)	0.2
Omentectomy, *n*/*n* total (%)	58/62 (94)	41/48 (85)	0.2
Appendectomy, *n*/*n* total (%)	44/62 (71)	31/48 (65)	0.5
Round ligament of the liver resection, *n*/*n* total (%)	47/62 (76)	32/48 (67)	0.3
Lesser omentum resection, *n*/*n* total (%)	13/62 (21)	8/48 (17)	0.6
Splenectomy, *n*/*n* total (%)	14/62 (23)	2/48 (4.2)	0.007
Pelvic peritonectomy, *n*/*n* total (%)	33/62 (53)	14/48 (29)	0.01
Diaphragmatic stripping, *n*/*n* total (%)	21/62 (34)	11/48 (23)	0.2
Cholecystectomy, *n*/*n* total (%)	3/62 (4.8)	0/48 (0)	0.3
Pelvic lymphadenectomy, *n*/*n* total (%)	14/62 (23)	9/48 (19)	0.6
Paraaortic lymphadenectomy, *n*/*n* total (%)	9/62 (15)	5/48 (10)	0.5
Pelvic exenteration, *n*/*n* total (%)	20/62 (32)	18/48 (38)	0.6
Colorectal resection, *n*/*n* total (%)	19/62 (31)	16/48 (33)	0.8
Hemicolectomy, *n*/*n* total (%)	2/62 (3.2)	2/48 (4.2)	>0.9
Pancolectomy, *n*/*n* total (%)	1/62 (1.6)	1/48 (2.1)	>0.9
Intestinal resection, *n*/*n* total (%)	4/62 (6.5)	4/48 (8.3)	0.7
Stoma, *n*/*n* total (%)	6/62 (9.7)	9/48 (19)	0.2
Intestinal anastomosis, *n*/*n* total (%)	15/62 (24)	10/48 (21)	0.7

**Table 6:** Changes in laboratory parameter values during the prehabilitation period.

Characteristic	Introductory Visit	Day of Admission to Hospital	Change *	*p*-Value
Hemoglobin concentration (g/dL), median [IQR]	12.8 (11.6, 13.7)	12.5 (11.3, 13.5)	−0.05 (−0.50, 0.30)	0.20
RBC, (×10^6^/µL), median [IQR]	4.4 (4.0, 4.7)	4.3 (3.9, 4.7)	0.00 (−0.10, 0.20)	0.55
WBC, (×10^3^/µL), median [IQR]	7.6 (6.0, 9.5)	7.9 (6.2, 9.7)	0.10 (−0.88, 1.35)	0.17
Total protein concentration (g/dL), median [IQR]	7.3 (7.0, 7.6)	7.30 (7.0, 7.5)	0.00 (−0.20, 0.18)	0.97
Albumin concentration (g/dL), median [IQR]	4.4 (4.2, 4.7)	4.40 (4.2, 4.7)	0.00 (−0.10, 0.20)	0.44
Creatinine, median [IQR]	0.80 (0.70, 0.90)	0.70 (0.70, 0.90)	0.00 (−0.10, 0.00)	0.12
Vitamin D concentration (ng/mL), median [IQR]	25 (16, 36)	38 (28, 46)	10 (4, 17)	<0.001

Abbreviations: IQR—interquartile range, RBC—red blood cells, WBC—white blood cells. *—The change was defined as the median difference between the value on the day of hospital admission and the initial visit.

**Table 7:** Comparison of the incidence of postoperative complications in group A and group B.

Characteristic	Group A (*n* Total = 62)	Group B (*n* Total = 48)	*p*-Value
ICU hospitalization, *n*/*n* total (%)	13/62 (21)	12/48 (25)	0.6
Number of days in ICU, median (IQR)	4.0 (3.0, 7.0)	5.0 (2.8, 8.0)	0.9
Reoperation, *n*/*n* total (%)	5/62 (8.1)	8/48 (17)	0.2
Wound dehiscence with eventration, *n*/*n* total (%)	3/62 (4.8)	6/48 (13)	0.2
Wound dehiscence without eventration, *n*/*n* total (%)	1/62 (1.6)	6/48 (13)	0.04
Need for vacuum therapy, *n*/*n* total (%)	1/62 (1.6)	6/48 (13)	0.04
Need for blood transfusion, *n*/*n* total (%)	14/62 (23)	20/48 (42%)	0.03
Need for parenteral nutrition, *n*/*n* total (%)	10/62 (16)	9/48 (19)	0.7
Readmission within 30 days, *n*/*n* total (%)	2/62 (3.2)	2/48 (4.2)	>0.9
Need for treatment in another department, *n*/*n* total (%)	0/62	5/48 (10)	0.01
Modification of antibiotic therapy due to increased inflammatory parameters, *n*/*n* total (%)	5/62 (8.1)	12/48 (25)	0.02
Postoperative respiratory failure requiring thoracentesis, *n*/*n* total (%)	2/62 (3.2)	3/48 (6.3)	0.7
Postoperative delirium, *n*/*n* total (%)	1/62 (1.6)	1/48 (2.1)	>0.9
Postoperative perforation of the urinary bladder, *n*/*n* total (%)	0/62	1/48 (2.1)	0.4
Death, *n*/*n* total (%)	1/62 (1.6)	2/48 (4.2)	0.58

Abbreviations: ICU—intensive care unit, IQR—interquartile range.

**Table 8:** Univariate and multivariate logistic regression analyses for postoperative complications.

Risk Factor	Univariate Logistic Regression		Multiple Logistic Regression	
Characteristic	OR (95% CI)	*p*-Value	OR (95% CI)	*p*-Value
Age (years)	1.02 (0.99, 1.07)	0.24		
Duration of surgery (min) [per 30 units]	1.40 (1.12, 1.79)	0.003	1.44 (1.13, 1.9)	0.006
FIGO				
1–2	Ref.			
3–4	1.99 (0.60–9.03)	0.28		
Haemoglobin (g/dL) ^1^	0.89 (0.65–1.19)	0.42		
Albumin concentration ^1^ (g/dL)	0.31 (0.1, 0.93)	0.04	0.82 (0.21, 3.34)	0.8
Total protein concentration ^1^ (g/dL)	0.3 (0.1, 0.77)	0.011	0.4 (0.11, 1.20)	0.13
Aletti complexity score	1.11 (0.94, 1.30)	0.2		
PCI	1.03 (0.98, 1.09)	0.23		
Group				
A	Ref.			
B	3.38 (1.33, 9.16)	0.01	3.31 (1.19, 9.93)	0.03
Duration of prehabilitation (days) [per 7 units]	0.8 (0.52, 1.04)	0.11		

Abbreviations: OR—odds ratio, CI—confidence interval, ^1^—measured on the day of admission to hospital (the day before surgery).

## Data Availability

Data are contained within the article.
